# Sexual Dimorphism of Metabolomic Profile in Arterial Hypertension

**DOI:** 10.1038/s41598-020-64329-1

**Published:** 2020-05-05

**Authors:** Yaya Goïta, Juan Manuel Chao de la Barca, Asmaou Keïta, Mamadou Bocary Diarra, Klétigui Casimir Dembélé, Floris Chabrun, Boubacar Sidiki Ibrahim Dramé, Yaya Kassogué, Mahamadou Diakité, Delphine Mirebeau-Prunier, Bakary Mamadou Cissé, Gilles Simard, Pascal Reynier

**Affiliations:** 1 Faculté de Pharmacie, Université des Sciences, des Techniques et des Technologies, de Bamako, Mali; 2 Service de Cardiologie, Centre Hospitalier Universitaire Mère-Enfant (CHUME) et Laboratoire d’analyses de Biologie médicale et Anatomo-Pathologique, Centre Hospitalier Universitaire Hôpital du Mali, Bamako, Mali; 3 Departement de Biochimie et Génétique, Centre Hospitalier Universitaire, Angers, France; 4 Unité Mixte de Recherche Mitovasc, Institut National de la Santé et de la Recherche Médicale (INSERM) U1083, Centre National de la Recherche Scientifique (CNRS) 6015, Université d’Angers, Angers, France; 5 Laboratoire d’analyses de Biologie médicale et Anatomo-Pathologique, Centre Hospitalier Universitaire Hôpital du Mali, Bamako, Mali

**Keywords:** Metabolomics, Predictive markers

## Abstract

Metabolomic studies have demonstrated the existence of biological signatures in blood of patients with arterial hypertension, but no study has hitherto reported the sexual dimorphism of these signatures. We compared the plasma metabolomic profiles of 28 individuals (13 women and 15 men) with essential arterial hypertension with those of a healthy control group (18 women and 18 men), using targeted metabolomics. Among the 188 metabolites explored, 152 were accurately measured. Supervised OPLS-DA (orthogonal partial least squares-discriminant analysis) showed good predictive performance for hypertension in both sexes (Q^2^cum = 0.59 in women and 0.60 in men) with low risk of overfitting (*p*-value-CV ANOVA = 0.004 in women and men). Seventy-five and 65 discriminant metabolites with a VIP (variable importance for the projection) greater than 1 were evidenced in women and men, respectively. Both sexes showed a considerable increase in phosphatidylcholines, a decrease in C16:0 with an increase in C28:1 lysophosphatidylcholines, an increase in sphingomyelins, as well as an increase of symmetric dimethylarginine (SDMA), acetyl-ornithine and hydroxyproline. Twenty-nine metabolites, involved in phospholipidic and cardiac remodeling, arginine/nitric oxide pathway and antihypertensive and insulin resistance mechanisms, discriminated the metabolic sexual dimorphism of hypertension. Our results highlight the importance of sexual dimorphism in arterial hypertension.

## Introduction

Arterial hypertension is defined by a systolic blood pressure greater than 140 mm Hg, a diastolic blood pressure greater than 90 mm Hg, or both^1^. Its multiple causes are often difficult to identify and the vast majority of cases are classified as essential hypertension. Hypertension affects about 1.13 billion people worldwide with prevalence above 20%^2,3^. Raised blood pressure is the greatest contributor to global mortality and may be responsible for 9.4 million deaths each year, mainly as the result of stroke and coronary heart disease [1].

In Africa, the prevalence of hypertension has greatly increased in recent decades, with the estimate in 2010 as high as 33.7%, representing 130.2 million cases^2,4,5^. In Mali, the prevalence of arterial hypertension was reported to vary from 21.1% in rural areas to 24.7% in urban areas^6^. It is well established that hypertension in people of African origin is more severe and develops earlier, with a higher percentage of complications than in the European population^4^. Recent studies showed that major determinants of hypertension in African adults are age, overweight, being of female sex, and that urban habitation likely provides a more obesogenic environment^6,7^. In addition, genetic factors that promote salt reabsorbtion also explain the frequency and severity of hypertension in African subjects^8^.

Several metabolomic studies have demonstrated the existence of biological signatures in the blood and urine of patients with high blood pressure from various regions, including African populations, and using various analytical platforms and study designs. These studies have been recently reviewed^9,10^. The metabolomic signatures published to date showed a wide variety of quantitative metabolite traits related to arterial hypertension such as amino acids (alanine, serine, glycine, methionine, arginine, tyrosine and isoleucine), nucleotide bases (adenine and uracil), hormones (epiandrosterone sulfate, 5 α-androstan-3β-diol disulfate, androsterone sulfate, melatonin, cortolone, dihydroxyphenylglycol, and hydroxyandrosterone), organic acids (hippurate, pyruvate, hexadecanedioate, formate, methyl nicotinate, dicarboxylic acids, butyrate and fumarate, lactate, malate and pyruvate), and hexoses^11–17^. A variety of lipids were also reported to be quantitatively modified in hypertension, such as phosphatidylcholines, ceramides, phosphatidylinositols, diacylglycerols, and fatty acids^14,16,18,19^. Changes in these signatures under the influence of dietary interventions in hypertensive patients (proline-betaine, carnitine, hippurate, cresyl sulfate, phenylacetylglutamine, N-methyl-2-pyridone-5-carboxyamide, methionine sulfone, and β-hydroxyisovalerate)^20,21^ or under various antihypertensive therapies (acylcarnitines, hexadecanedioate uric acid, lysophosphatidylcholines, triacylglycerols, and cholesterol esters) have also been reported^22–24^.

The pathophysiology of essential hypertension is strongly influenced by sex. Indeed, sex hormones such as estrogens, the metabolism of nitric oxide (NO), the renin-angiotensin-aldosterone system, the immune system, kidney function, and sodium transport, which are factors controlling the blood pressure, all present differences according to sex^25,26^. Men have higher blood pressure than pre-menopausal women and are therefore more prone to hypertension. However, after the menopause the incidence of hypertension is four times higher in women, while it is only three times higher in age-matched men^27^. Finally, the effect of antihypertensive treatments is also known to depend to a large extent on sex^28^.

Despite a clear sexual dimorphism in the pathophysiology of hypertension, to our knowledge no study has hitherto presented the sexual dimorphism of metabolomic signatures. Therefore, we carried out the present study applying a highly standardized, targeted, metabolomic approach in order to compare the plasma of hypertensive individuals from Bamako, Mali according to sex.

## Methods

### Ethics statement

**Figure 1:**
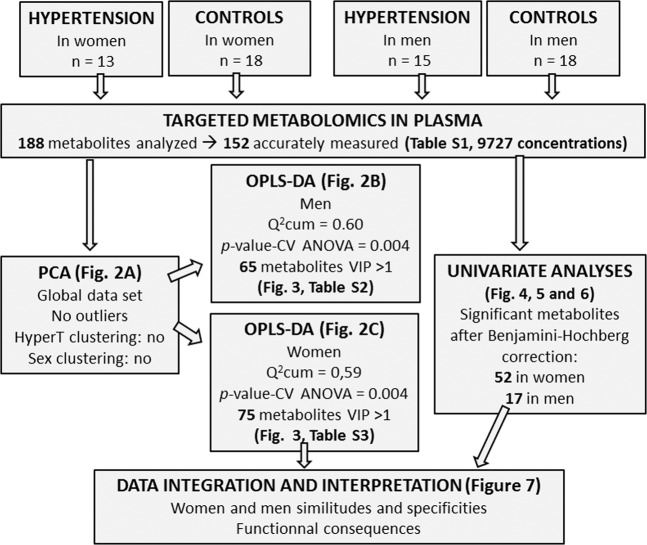
Global flow chart of the study design.

Figure 1 shows the global flow chart of the study design. The study was conducted in accordance with the Declaration of Helsinki (1983) and was approved by the Ethical Review Committee of the Faculty of Medicine, Pharmacy and Odonto-Stomatology (FMPOS) of the University of Technical and Technological Sciences of Bamako (USTTB) in Mali, under the reference 2016/05/EC/FMPOS of 18^th^ January 2016. Participants were included after having given their informed written consent.

### Study participants

The study included 64 individuals aged from 34 to 60 years, of which 28 were hypertensive (13 women and 15 men) and 36 were non-hypertensive (18 women and 18 men). All hypertensive subjects included in the study were seen in the Department of Cardiology the University Hospital Le Luxembourg of Bamako (Centre Hospitalier Universitaire Mère-Enfant, CHUME) and had a verified blood pressure at rest of above 140/90 mm Hg. Control subjects were selected from healthy individuals accompanying the patients at the University Hospital. Their inclusion criteria were the verified absence of high blood pressure (<140/80 mm Hg) and the absence of any other cardiovascular disease. The recruitment of participants was carried out from October 2017 to March 2018.

### Sample collection

Blood samples were taken, in the morning after 12 hours of fasting, collected in heparin tubes and centrifuged immediately for 5 minutes at 3000 g at +4 °C before plasma recovery. The plasma was then stored at −80 °C in one ml aliquots and transported on ice to the Biochemistry Laboratory of the University of Angers, France, where the metabolomic analysis was performed.

### Metabolomics analysis

Targeted quantitative metabolomics analyses were performed using the Biocrates Absolute IDQ p180 kit (Biocrates Life Sciences AG, Innsbruck, Austria) as described previously^29^. Used with mass spectrometry (QTRAP 5500; SCIEX, Villebon-sur-Yvette, France), this kit allows quantification of up to 188 different endogenous molecules distributed as follows: free carnitine (C0), 39 acylcarnitines (C), the sum of hexoses (H1), 21 amino acids, 21 biogenic amines, and 105 lipids. The lipids are distributed in the kit in four different classes: 14 lysophosphatidylcholines (lysoPC), 38 diacyl phosphatidylcholines (PC aa), 38 acyl-alkylphosphatidylcholines (PC ae), and 15 sphingomyelins (SM). Flow injection analysis, coupled with tandem mass spectrometry (FIA-MS/MS), was used for analysis of carnitine, acylcarnitines, lipids, and hexoses. Liquid chromatography (LC) was used for separating amino acids and biogenic amines before quantitation with mass spectrometry.

All reagents used in this analysis were of LC-MS grade and purchased from VWR (Fontenay-sous-Bois, France) and Merck (Molsheim, France). Sample preparation and analysis were performed following the Biocrates Kit User Manual^30^. Each plasma sample was thoroughly vortexed after thawing and centrifuged at +4 °C for 5 minutes at 5000 g. Then, 10 microliters of each sample were added to the filter on the upper wells of the 96-well plate. Metabolites were extracted and derivatized for quantitation of amino acids and biogenic amines. Lastly, the extracts were diluted with MS running solvent before FIA- and LC-MS/MS analysis. Three quality controls (QCs) composed of human plasma samples at three concentration levels of low (QC1), medium (QC2), and high (QC3) were used to evaluate performance of the analytical assay. A seven-point serial dilution of calibrators was added to the 96-well plate of the kit to generate calibration curves for quantification of amino acids and biogenic amines.

### Statistical analyses

Univariate analysis of clinical data was performed using the bilateral Student’s t-test, with differences considered significant at P-value < 0.05.

Multivariate analyses were first performed with SIMCA-P v.14.1 (Umetrics, Umeå, Sweden) as described previously^29^. Unsupervised principal component analysis (PCA) was used for the detection of outliers and spontaneous sample grouping. Supervised orthogonal partial least squares discriminant analysis (OPLS-DA) was then applied to maximize variation between hypertensive and control groups, and to determine the metabolites contributing to this variation. PCA and OPLS-DA models were performed on mean-centered and unit variance-scaled (MC-UV) data.

The quality of the OPLS-DA model was validated by two parameters: the goodness of fit (*R*^2^) and the goodness of prediction indicated by the cumulated Q^2^ value (Q^2^cum). A threshold of 0.5 was used to determine whether an OPLS-DA model could be estimated as having a good (Q^2^cum ≥ 0.5) or poor (Q^2^cum < 0.5) predictive capability^31^. VIP (variable importance for the projection) values summarized the importance of each variable for the OPLS-DA model, whereas the loading values were indicators of the relationship between the **y** vector containing the class information (i.e. Hypertension or Control) and variables in the **X** matrix (i.e. metabolites). Variables with a VIP value greater than unity were considered as important for group discrimination in predictive models^31^. The risk of overfitting and the robustness of the final reduced model were assessed by the intercept of the permutation plot (permQ_2_) and cross-validated analysis of variance (CV-ANOVA). Non-over-fitted models have a negative permQ_2_ intercept and are considered significantly different (P-value < 0.05) from models obtained by randomly permutating the **y** vector only whilst the rows of the associated **X** matrix are not permutated. In a case in which a model with good predictive capabilities is found, variable selection is based on VIP and loading values scaled as correlation coefficients (p_corr_). In order to make it easier to visualize of important variables for group discrimination, we built a word cloud. In the word cloud each word represents the corresponding metabolite with word size as a function of the VIP, whilst loading determines word color intensity.

Univariate analysis of metabolomics data was performed using the Student’s t test of log transformed data. The Benjamini-Hochberg (BH) correction was used to compare multiple metabolite concentrations to keep the false discovery rate (FDR) < 5%. After BH correction results were presented as a volcano plot with fold change between hypertensive and normotensive individuals for each metabolite in the x-axis and the P-value in the y-axis. As far as P-values are in the interval [0.1] and that significance increases with the decreasing of the P-value, we represented the logarithm of the P-value instead of the P-value itself using the last significant P-value after BH correction as the base of this logarithm. This way, significant variables (i.e., metabolites) after BH correction have log-transformed P-values greater than one.

### Arginine/ornithine and PC ae/PC aa ratios

The arginine/ornithine ratio, reflecting the plasma arginase activity in hypertensive women and men, was calculated from the average plasma concentrations of arginine and ornithine. The PC ae/PC aa ratio in hypertensive women and men was calculated using the average plasma concentrations of diacyl-phosphatidylcholines (PC aa) and acyl-alkyl-phosphatidylcholines (PC ae).

## Results

### Clinical features of participants

Table 1 presents comparisons of clinical data of individuals with arterial hypertension (n = 13 women and n = 15 men) and controls (n = 18 women and n = 18 men) stratified by sex. The mean age of individuals with hypertension did not differ significantly from that of the controls. There was a difference between groups in systolic and diastolic blood pressure, in both women and men, as well as in body mass index (BMI) and homocysteinemia in women only. There were no differences between groups for diabetes, renal filtration, inflammation, lipid profile and vitamin B12. The antihypertensive treatments taken by patients are listed in Table 2.

**Table 1:** Clinical data of hypertensive and control groups stratified by sex.

Clinical data (medians)	Men (n = 33)			Women (n = 31)		
	hypertensive n = 15	controls n = 18	*p*	hypertensive n = 13	controls n = 18	P-value
Age (years)	47 (36–55)	40 (36–58)	*0.103*	46 (34–51)	40.5 (35–60)	*0.46*
SBP (mm Hg)	170 (150–180)	120 (110–130)	3E-15*	170 (160–180)	120 (100–130)	2E-15*
DBP (mm Hg)	110 (100–120)	80 (70–80)	2E-15*	110 (110–120)	80 (70–80)	6E-19*
BMI (kg/m²)	26 (16–34)	22 (18–35)	*0.098*	29 (24–35)	21.5 (17–24)	4E-9*
Gly (mmol/L)	4 (2–5.5)	4.7 (4.0–6.0)	*0.050*	4.8 (3.0–11.8)	4.4 (3.6–6.0)	*0 0.47*
GFR (mL/mn)	106 (25–155)	110 (85–156)	*0.366*	116 (4–188)	142 (87–205)	*0.168*
Hcyst (µmol/L)	20 (16–78)	18 (12–50)	*0.104*	16 (11–52)	13.5 (9–24)	0.049*
NT-PBNP (ng/L)	97 (35–8522)	35 (35–116)	*0.196*	70 (35–5850)	52.5 (35–276)	*0.102*
CRP*us* (mg/L)	2.9 (0.2–12.9)	0.9 (0.2–6.2)	*0.078*	4.4 (0.2–23.9)	1.8 (0.2–9.1)	*0.116*
Chol (mmol/L)	3.9 (2.4–6.2)	4.2 (2.9–5.6)	*0.710*	4.3 (2.8–7.4)	4.1 (3.1–6.3)	*0.440*
HDL (mmol/L)	1.1 (0.5–1.6)	1 (0.55–1.47)	*0.491*	1.1 (0.8–2)	1.15 (0.9–1.4)	*0.976*
LDL (mmol/L)	2.4 (1.3–4.4)	2.65 (1.5–3.6)	*0.918*	2.7 (1.5–5.2)	2.4 (1.6–4.7)	*0.458*
TG (mmol/L)	0.8 (0.37–1.68)	0.91 (0.45–3.56)	*0.203*	0.81 (0.43–2,1)	0.85 (0.33–1.51)	*0.524*
ApoA1 (g/L)	1.2 (0.69–1.49)	1.18 (0.91–1.3)	*0.898*	1.26 (1.07–1.62)	1.21 (0.64–1.46)	*0.059*
ApoB (g/L)	0.83 (0.56–1.47)	0.78 (0.45–1.09)	*0.505*	0.91 (0.48–1.81)	0.75 (0.48–1.35)	*0.292*
Lp(a) (g/L)	0.2 (0.1–0.85)	0.2 (0.1–0.8)	*0.457*	0.2 (0.1–0.6)	0.2 (0.10–0.5)	*0.371*
VITB12	523 (220–815)	417.5 (239–833)	*0.153*	370 (268–741)	505 (183–770)	*0.557*

SBP: systolic blood pressure; DBP: diastolic blood pressure; BMI: body mass index; Gly: glycemia; GFR: glomerular filtration rate; Hcyst: homocysteinemia; NT-PBNP: Pro-Brain Natriuretic Peptide; CRP*us*: ultrasensitive C-Reactive Protein; Chol: total cholesterol; GFR: glomerular filtration rate; TG: triacylglycerols, HDL: HDL-cholesterol; LDL: LDL-cholesterol; TG: triacylglycerols; ApoA1: Apoprotein A1; ApoB: Apoprotein B; Lp(a): Lipoprotein a; VITB12: vitamin B12. * P-value < 0.05.

**Table 2:** Antihypertensive treatments.

Treatments	Men with hypertension n = 15	Women with hypertension n = 13
Low sodium diet	15 (100%)	13 (100%)
HTA treatments	9 (60%)	13 (100%)
Diuretic (furosemide)	4 (26.66%)	2 (15.38%)
Calcium channel blocker (atenolol, nicardipine, amlodipine, nifedipine)	5 (33.33%)	4 (30.76%)
Beta blocker (atenolol)	1 (6.66%)	2 (15.38%)
Antiarrhythmic (amiodarone, cardiurine)	2 (13.33%)	1 (7.69%)
Central antihypertensive (methyldopa)	0 (0%)	5 (38.46%)
Traditional medicine (plants)	5 (33.33%)	0 (0%)

### Metabolomics analysis

Before statistical analysis, the raw metabolomics data were examined to exclude metabolites with >20% concentration values below the lower limit of quantitation (LLOQ) or above the upper limit of quantitation (ULOQ). After validation of the kit plate based on quality control (QC) samples, 36 (19%) metabolites were excluded because of inaccurate measurements. The statistical analysis was then performed on the 152 (81%) remaining metabolites (Supplementary Table S1).

### Multivariate analyses

**Figure 2:**
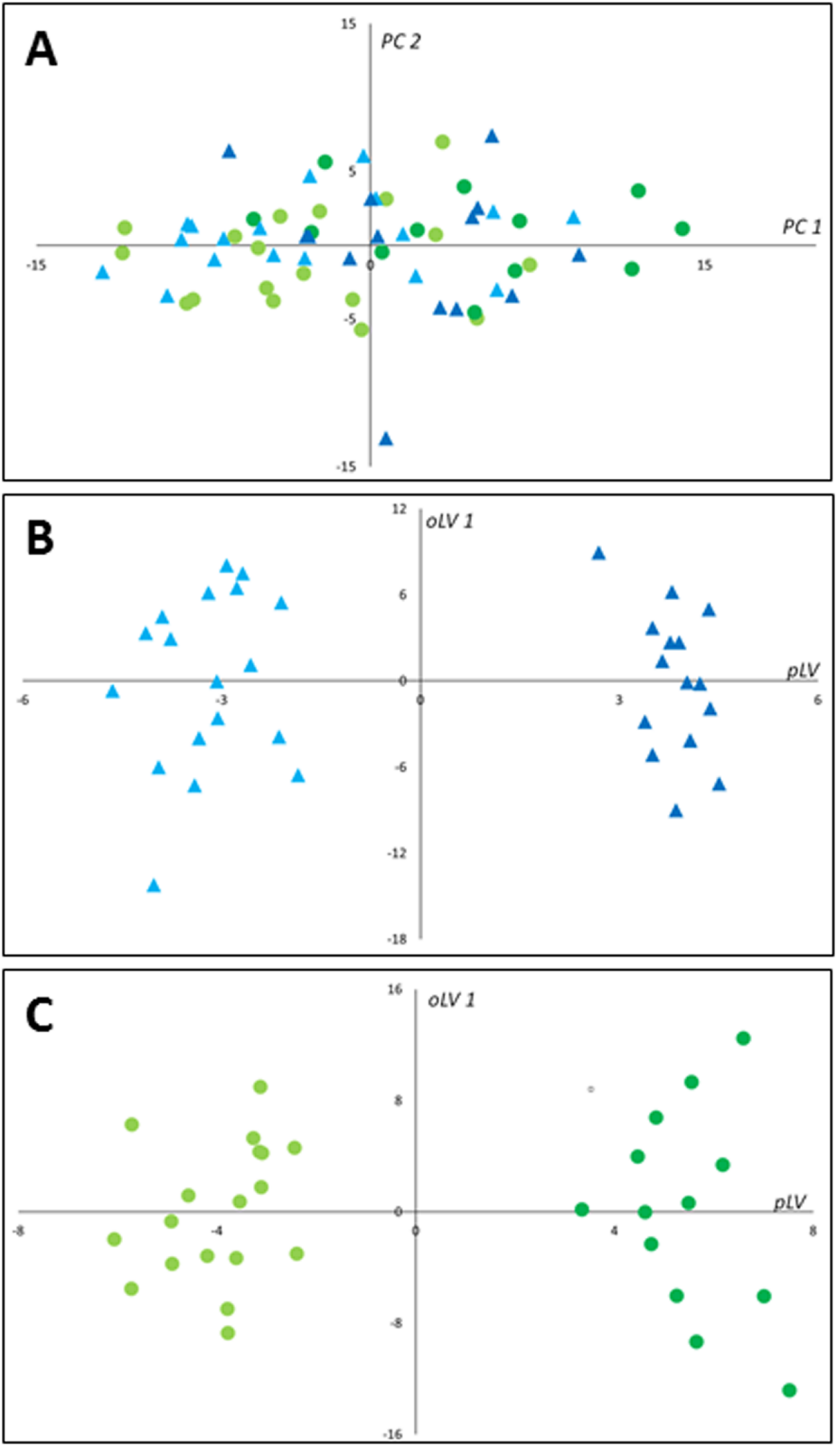
Scatter plots of multivariate analyses of metabolomics data. (**A**) First principal plan of the PCA performed on the global data set. No spontaneous grouping is evidenced; (**B**) Supervised OPLS-DA comparing hypertensive patients to controls in men with an overt separation of both groups; (**C**) Supervised OPLS-DA comparing hypertensive patients to controls in women showing similar good discrimination level between compared groups. The OPLS-DA models in both men and women provided a good fit for the data (R^2^Y = 0.97 in men and 0.95 in women), good predictive capability (Q^2^cum = 0.6 in men and 0.59 in women) and low risk of over-fitting (permQ^2^ = −0.72, P-value CV-ANOVA = 0.009 in men and permQ^2^ = −0.66, P-value CV-ANOVA = 0.005 in women). PC1, 2: first and second principal components; pLV: predictive latent variable; oLV 1: first orthogonal latent variable.

Principal component analysis scatter plot of the whole data did not show any spontaneous grouping according to arterial hypertension status or sex, nor did it show any strong outliers (Fig. 2A).

**Figure 3:**
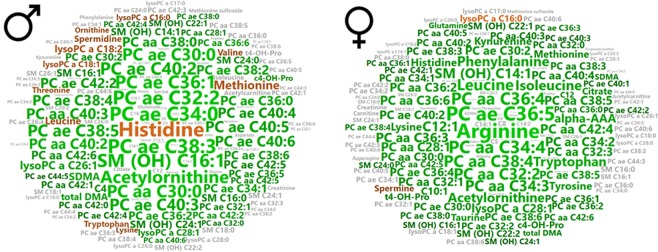
Word cloud visualization of the plasma metabolomic signature of hypertension in men and women. Word clouds were computed using metabolite VIPs to determine the size of the labels, and loadings to determine the color scale. Metabolites with negative loadings values (decreased in hypertension) are presented in brown, metabolites with positive loadings values (increased in hypertension) in green. Metabolites with a VIP < 1 are shown in gray.

In men, the OPLS-DA supervised method revealed a clear discrimination with good predictive capability and a low risk of over-fitting between the hypertensive and control groups (Q^2^cum = 0.6; permQ^2^ = −0.72, P-value CV-ANOVA = 0.009, Fig. 2B). The best discriminant metabolites (with VIP ≥ 1 and high absolute p_corr_ values) contributing to the model included a subset of 65 (~43%) of the accurately measured metabolites in the hypertension group, comprising 13 amino acids and biogenic amines (total dimethyl-arginine, symmetric dimethyl-arginine, histidine, methionine, leucine, tryptophan, threonine, spermidine, valine, ornithine, acetyl-ornithine, lysine and c4-OH-Pro), one acylcarnitine (C4), 39 phosphatidylcholines, five lysophosphatidylcholines and seven sphingomyelins. Among these discriminant metabolites 12 (histidine, leucine, tryptophan, methionine, valine, ornithine, lysine, spermidine and three lysophosphatidylcholines) showed reduced concentrations in patients with hypertension when compared to control groups; the other 53 metabolites had all an increased concentration in the hypertensive group. These discriminant metabolites in men are presented in Supplementary Table S2 and Fig. 3.

In women, the OPLS-DA supervised method revealed a clear discrimination with good predictive capability and low risk of over-fitting between the hypertensive and control groups (Q^2^cum = 0.59; permQ^2^ = −0.66, P-value CV-ANOVA = 0.005, Fig. 2C). The best discriminant metabolites (with VIP ≥ 1 and high absolute p_corr_ values) contributing to the mode included a subset of 75 (49%) of the accurately measured metabolites in the hypertension group, comprising 20 amino acids and biogenic amines (arginine, total dimethyl-arginine, symmetric dimethyl-arginine, acetyl-ornithine, trans- and cis- hydroxyproline stereoisomers (t4-OH-Pro and c4-OH-Pro), glutamine, methionine, citrulline, lysine, histidine, tyrosine, leucine, isoleucine, phenylalanine, tryptophan, kynurenine, spermine, taurine and alpha-aminoadipate), three acylcarnitines (C12:0, C10:1 and C12:1), 44 phosphatidylcholines, two lysophosphatidylcholines, and six sphingomyelins. Among these 75 discriminant metabolites only two metabolites (spermine, LysoPC a C16:0) showed reduced concentrations in patients with hypertension when compared to control groups; the other 73 metabolites were all increased in the hypertensive group. These discriminant metabolites in women are presented in Supplementary Table S3 and in Fig. 3.

### Univariate analyses

**Figure 4:**
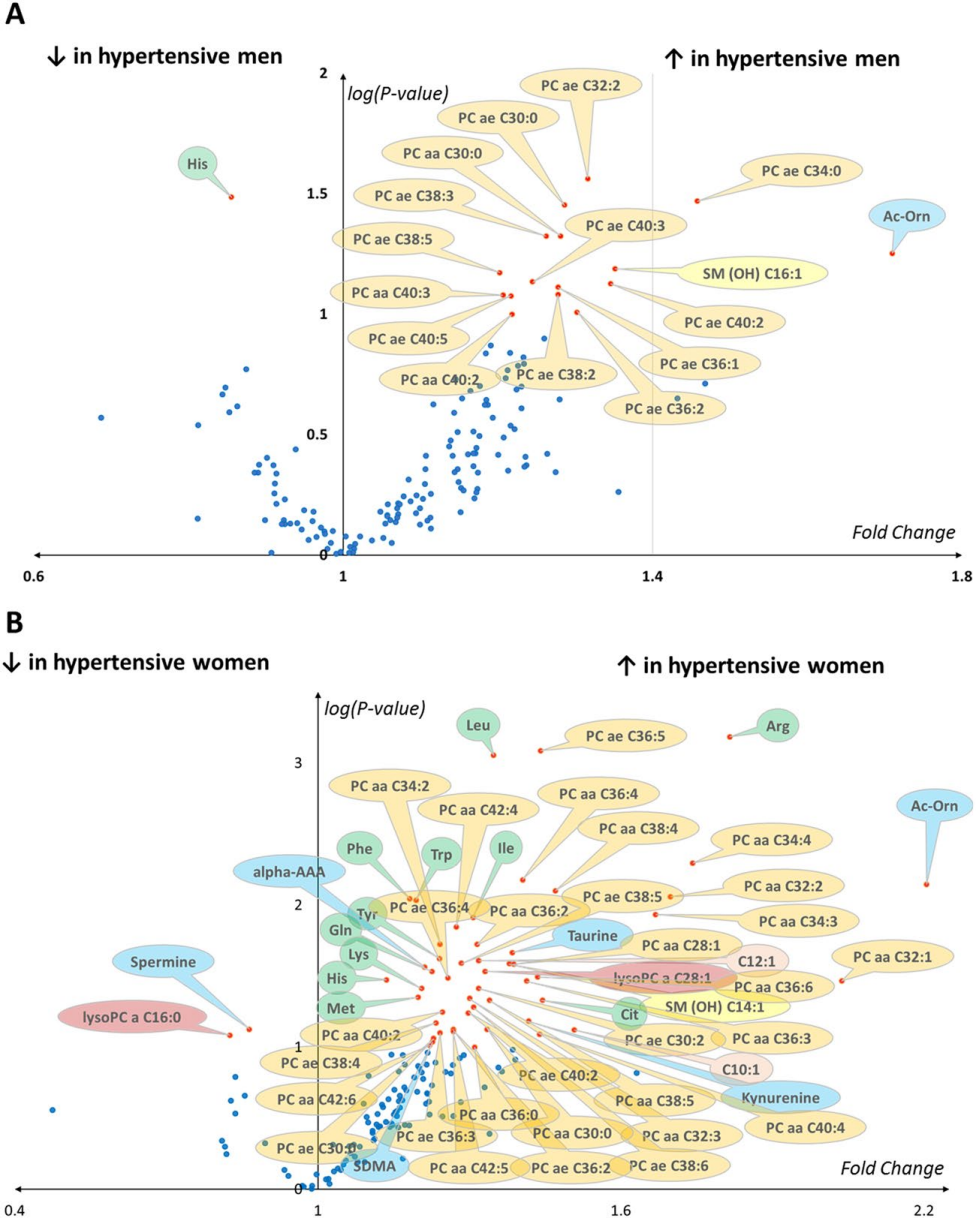
Results of the univariate analysis for men (**A**) and women (**B**) represented as volcano plots. The x-axis represents fold changes for the mean of each metabolite concentration between hypertensive and normotensive individuals. Log-transformed P-values (log(P-value)) obtained after Student’s t-test between hypertensive and normotensive groups are contained in the y-axis using the greater retained P-values after BH correction (0.0064 and 0.019) as bases for the logarithms of P-values in men and women, repsectively. Only significantly different metabolites after BH correction (red dots) have log-transformed P-values ≥ 1 and are labelled. In lysophosphatidylcholines, hydroxysphingomyelins, phosphatidylcholines and acylcarnitines the length and the degree of unsaturation of the acyl moieties are separated by a colon and appear after the capital letter “C” in the short name of these molecules. Non-significant metabolites after BH correction appear as blue dots and are not labelled. Bubbles used to spot metabolites are colored according to the biochemical family as follows: light brown for acylcarnitines; green for amino acids; blue for biogenic amines; dark brown for lysophosphatidylcholines; orange for phosphatidylcholines and yellow for sphingomyelin.

After Benjamini-Hochberg correction, 17 metabolites were significant in hypertensive men compared to controls (Fig. 4A). Among these 17 significant metabolites, 16 were included in the 65 metabolites with a VIP > 1 found in the multivariate model. In hypertensive women, 52 metabolites were significant after Benjamini-Hochberg correction, all of them being included in the 75 metabolites with a VIP > 1 found in the multivariate model (Fig. 4B).

In men, histidine was the only metabolite significantly decreased in hypertensive patients, whilst acetyl-ornithine (Ac-Orn) and 15 lipids including hydroxysphingomyelin 16:1 (SM(OH) C16:1), 3 diacyl phosphatidylcholines (PC aa) and 11 alkyl-acyl phosphatidylcholines (PC ae) were significantly increased in men suffering from hypertension.

In women, high-order polyamine spermine and lysophosphatidylcholine 16:0 (lysoPC a C16:0) were found to be decreased in hypertensive patients, whilst 50 metabolites were increased including 11 amino acids (arginine, leucine, isoleucine, tryptophan, phenylalanine, tyrosine, lysine, glutamine, histidine, methionine, and citrulline), 5 amino acid-derived metabolites (kynurenine, acetyl-ornithine, α-aminoadipic acid, symmetric dimethylarginine, and taurine), two medium-chain monounsaturated acyl-L-carnitines (decenoyl-L-carnitine or C10:1 and dodecenoyl-L-carnitine or C12:1), one hydroxy-sphingomyelin (SM(OH) C14:1), one lysophosphatidylcholine (lysoPC a C28:1) and 30 phosphatidylcholines including 20 diacyl phosphatidylcholines (PC aa) and 10 alkyl-acyl phosphatidylcholines (PC ae).

**Figure 5:**
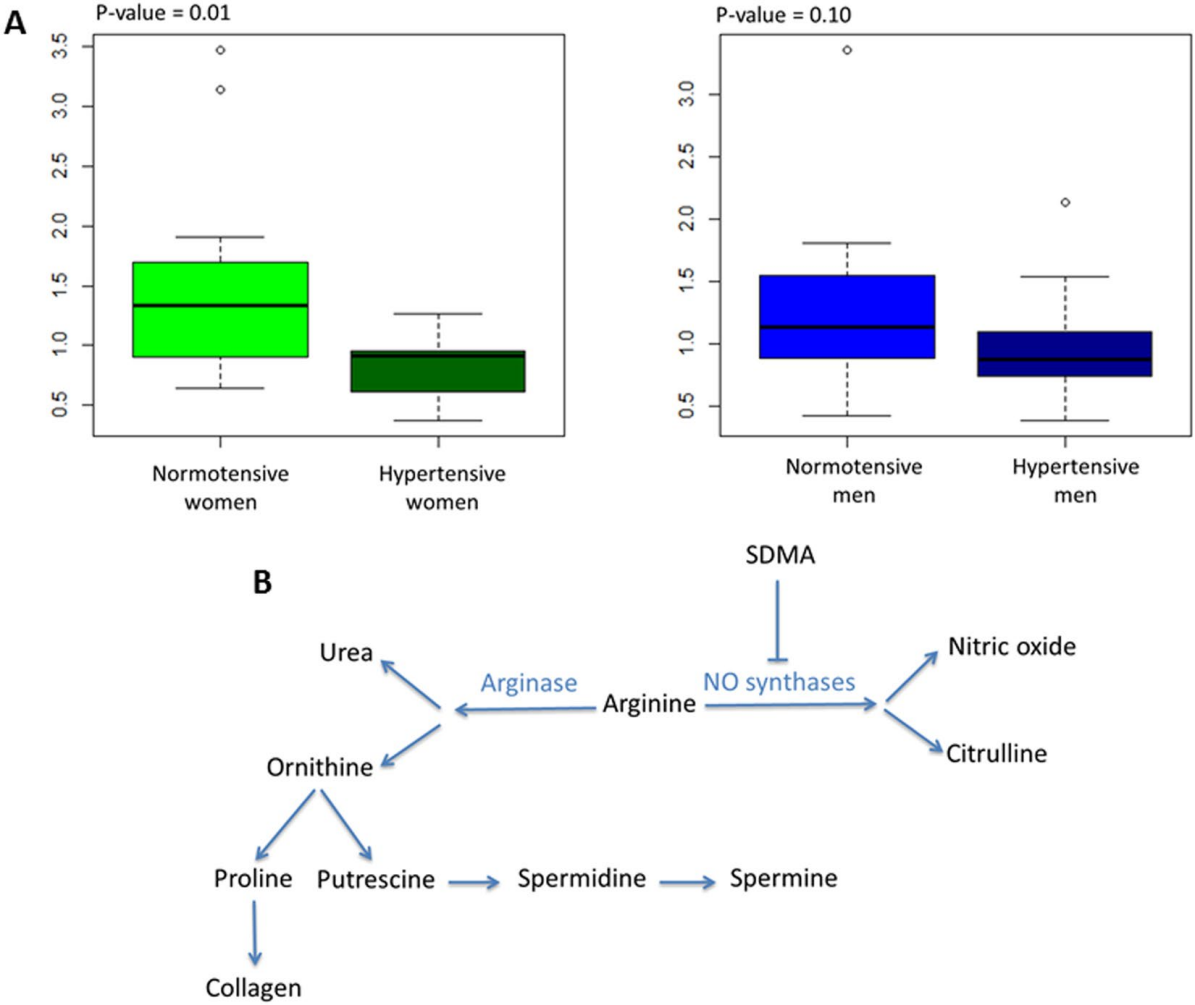
Arginine/ornithine ratio. (**A**) Arginase activity, in hypertensive men and women, was calculated using the ratio of the average plasma concentrations of arginine/ornithine. (**B**) Arginine and ornithine metabolic pathways.

**Figure 6:**
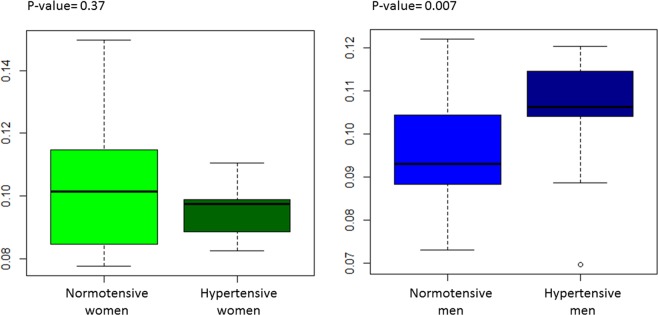
PC ae/PC aa ratio. The PC ae/PC aa ratio in hypertensive men and women was calculated from the average plasma concentrations of diacyl-phosphatidylcholines (PC aa) and acyl-alkyl-phosphatidylcholines (PC ae).

The arginine/ornithine ratio, providing the arginase activity, was significantly reduced in hypertensive women compared to their controls, whereas it was not significantly affected in men (Fig. 5A). The PC ae/PC aa ratio was significantly increased in hypertensive men compared to the controls, whereas it was not significantly affected in women (Fig. 6).

## Discussion

The multivariate models show 103 metabolite variations of concentration in the plasma that are involved in the biological imprinting of hypertension, 34 (33%) being common to both sexes, 40 (39%) being specifically modified in women and 29 (28%) in men.

A significant part of the signature is represented by a massive increase in the phosphatidylcholine concentrations (n = 39 in men and n = 44 in women) in the hypertensive subjects, 23 of these phosphatidylcholines being chemically identical in both sexes. In addition, two lysophosphatidylcholines, which are the result of phosphatidylcholine hydrolysis by phospholipase A2, were modified (decreased LysoPC C16:0 and increased LysoPC a C28:1) in hypertensive patients of both sexes.

Several studies have found changes in phosphatidylcholine concentrations in the blood of hypertensive patients. The increase of two acyl-alkyl phosphatidylcholines has been associated with an increased risk of developing hypertension in a prospective cohort [14]. The increased concentration of three phosphatidylcholines has also been reported in a British hypertensive population^19^. This study also revealed a disruption of lysophosphatidylcholines, highlighting their potential role in inflammation. The concentration of blood phosphatidylcholines may be related to several comorbidities of hypertension, such as body mass index, dyslipidemia, and insulin resistance^32^. Our study appears to be the first to identify such a massive increase in the global content of these phosphatidylcholines in hypertension and to show that this biochemical phenotype is globally common to both sexes. However, a characteristic that specifically affects hypertensive men and not women is the overrepresentation of the acyl-alkyl forms of phosphatidylcholines. These molecules, previously defined as plasmalogens, disclose antioxidant properties attributed to the vinyl ether bond that reacts with reactive oxygen species^33^. In the absence of the protective effect of estrogens, men are more exposed than women to oxidative stress. The increased concentration of plasmalogens in men could therefore represent an alternative anti-oxidant system. Lysophosphatidylcholines have also been found to be modified in the plasma of pre-hypertensive patients^23^, but it should be noted that, in contrast to our own findings, in this study the LysoPC C16:0 was seen to be at higher level.

Six sphingomyelins have an increased incidence in hypertensive women and seven in men, five of which are common to both sexes. Research literature to date has already reported a modification of sphingomyelin concentrations in the plasma of a cohort of hypertensive men with an increase of SM C16:1 and SM C24:2^22,34^. Notably, we also found this increase of SM C16:1 in both sexes. Such alteration of the sphingomyelin profile has also already been reported in preeclampsia^35,36^ and it has been identified elsewhere that red blood cell membranes in hypertensive rats have a higher sphingomyelin content, thus explaining their reduced membrane fluidity^37^. Sphingomyelins may also be involved in hypertension, given their correlation either with insulin resistance or with inflammation^38^.

Another notable common characteristic in both sexes is the increased concentration of hydroxyproline. An increased hydroxyproline concentration has been shown in the urine of patients with primary aldosteronism that may contribute to hypertension^39^. But the increased hydroxyproline in this study was sex-specific, affecting only women, while it is present in both sexes in our study. This increased hydroxyproline most likely results from cardiac remodeling in response to high blood pressure, since hydroxyproline is a biomarker of collagen turnover that was shown to be increased in the left ventricle of a rat model made hypertensive by the administration of L-NAME^40^.

Further noteworthy features common to both sexes are the increase in the concentration of symmetric dimethylarginine (SDMA) and total dimethylarginine (total DMA), the asymmetric dimethylarginine (ADMA) being accurately measured but not significantly modified. ADMA and SDMA are both produced by tissue degradation through the hydrolysis of arginine-containing endogenous proteins that may result from the same mechanism of protein turnover leading to increased hydroxyproline. Their structural similitude with arginine interferes with arginine-related signaling, transport and uptake. The involvement of ADMA in hypertension, through its inhibitory effect on nitric oxide (NO) synthesis, is well established^41^, whereas it is less clear in the case of SDMA^42^. However, both ADMA and SDMA reduce the NO bioavailability, whereby SDMA reduces the endothelial NO synthesis through limiting the arginine supply to NO synthase^43,44^. The increase in both sexes of SDMA, as well as total DMA, is a pejorative sign of cardiovascular risk, since a meta-analysis showed that ADMA and SDMA are independent risk markers for cardiovascular mortality^45,46^. SDMA is also a good marker of kidney failure, but there was no difference in creatinine concentrations in our hypertensive and control populations.

The increased SDMA and total DMA concentrations in our study affect both sexes but other metabolites related to arginine pathway, such as arginine and ornithine (Fig. 5B) exhibit sexual dimorphism. The basic pathophysiological mechanism could therefore be globally the same in women and men but with subtle variations according to sex. Arginine and its precursor citrulline were only increased in women; the arginine product ornithine was only decreased in men and its precursor acetyl-ornithine was increased in both sexes. Interestingly, the increased concentrations of hydroxyproline and acetyl-ornithine have been previously reported in a rat model of pulmonary arterial hypertension^47^. As shown in Fig. 3, the increased arginine in women is an important feature of the signature with a high VIP, as well as the increased acetyl-ornithine in both sexes. SDMA limiting the arginine supply to NO synthase could explain the increased concentration of arginine in the blood of women. Spermidine was reduced only in men whereas its direct product spermine was only reduced in women. Interestingly, all these metabolites belong to a common metabolic pathway connected to the arginase activity that directs the arginine either toward the synthesis of NO or towards that of ornithine. Ornithine itself can be directed either towards the synthesis of spermine and spermidine polyamines or towards proline synthesis which is also affected in our signature as seen above with regard to hydroxyproline and collagen turnover. The arginine/ornithine ratio, reflecting the arginase activity, demonstrates this sexual dimorphism because it is lowered in hypertensive women and unmodified in men.

It should be noted that this ratio has already been reported as lowered in patients with pulmonary arterial hypertension, but in both men and women^47^. Moreover, spermidine feeding was shown to reduce systemic blood pressure and to delay the progression to heart failure in a rat model; in humans, the same study provided evidence that high levels of dietary spermidine, assessed from food questionnaires, had a high correlation with reduced blood pressure and lower incidence of cardiovascular disease^48^.

Eleven amino acids (arginine, citrulline, tryptophan, glutamine, histidine, tyrosine, leucine, isoleucine, phenylalanine, methionine and lysine), three biogenic amines (kynurenine, taurine, alpha-aminolipate and spermine), and three acylcarnitines (C10, C12 and C12:1 = dodecanoylcarnitine and decenoylcarnitine) are specifically increased in hypertensive women. The increased level of glutamine may be related to the increased hydroxyproline because it is the precursor of the proline biosynthesis, an essential element of collagen. Interestingly, four of these amino acids or biogenic amines have already been reported to display antihypertensive properties, as demonstrated for histidine through central histamine H3 receptors and decreased NO^49^, for tyrosine through the effect of its product, norepinephrine, on the central nervous system^50^, for phenylalanine through GTP cyclohydrolase inhibition^51^, and for taurine^52^. These discriminant metabolites may represent protective mechanisms to counteract increased blood pressure in women. These protective metabolites are not increased in men and histidine, the metabolite with the highest VIP (Fig. 3) of the male signature, is even diminished in men. Kynurenine is known to be involved in renovascular hypertension^53^ and in arterial hypertension and it could exert a protective vasodilatory effect through the NO pathway^54^. Intriguingly, the tryptophan that is the precursor of kynurenine shows inverse variation in women and men with an increased concentration in women, consistent with the elevation of kynurenine, while it is lowered in men. The increased concentration of three acylcarnitines in women is known as a risk factor of cardiovascular death^55^. Taken together, the increased branch chain amino acids (leucine, isoleucine), acyl-carnitines, and alpha-aminoadipate are typical of an insulin resistance signature^56^ that is found in women, and these biomarkers themselves are not present in hypertensive men. Indeed, only one acylcarnitine (C4 = butyrylcarnitine) was specifically increased in men, and the branched amino acids leucine and valine were somewhat lower in men. The presence of this signature of insulin resistance in women in our cohort, despite the absence of proven diabetes, is probably related to the fact that the group of hypertensive female subjects tended to exhibit different BMI profile when compared with their control group, while no such difference existed between the two classes of male subjects. The fact that hypertensive women in our cohorts have a significantly different BMI from that of the controls is therefore a limit of our study, since the insulin resistance signature found in women could be at least partially linked to this difference in BMI. Elevated concentrations of branched amino acids are known to promote endothelial dysfunction, inflammation, and oxidative stress^57^ and may promote hypertension^58^. Insulin resistance could contribute to hypertension through altered NO release^59^. Other specificities of hypertension in men were the decreased concentrations of threonine and lysoPC a C26:1, as well as the reduced concentrations of two amino acids (methionine and lysine) that are rather increased in hypertensive women. The fact that the treatments taken by hypertensive men and women in our study were not strictly comparable, thus presenting another limitation. Indeed, the differences in metabolic profiles between these two groups may have been influenced by their respective treatments.

Biological sex is known to have a significant impact on the development of hypertension^25,26^. Significant disparities between the sexes have been shown in NO pathways, the renin-angiotensin system, the sympathetic nervous system, inflammation, and renal function, suggesting important sexual differences in the control of blood pressure. Sex hormones, sex chromosomes, and lifestyle related to gender differences thus contribute to this dimorphism of hypertension. Understanding this dimorphism will be crucial in future efforts to better personalize the prevention and treatment of increased blood pressure^60^. Our study shows that all these dimorphic mechanisms leave their biological imprinting in the blood. For example, the sexual dimorphism of insulin resistance through estrogen receptors is established [56] and insulin resistance plays a role in the arginine/NO pathway^15^. These two features (insulin resistance and altered arginine/NO pathway) may explain much of the sexual dimorphism of the hypertension metabolomic signature observed in this study. Quite in line with our own results presented here, further research has identified that in different African populations from different origins, 43% of the difference in hypertension prevalence for women was attributable to obesity, and only 22% for men, whereas respective values for obesity were 14% and 11%, indicating a strong link between adiposity and hypertension in African women across a variety of environments^61^. It would therefore be interesting to replicate a metabolomic study like this one, but looking at larger populations and at different stages in sexual dimorphism (pre-puberty, reproductive age, and post-menopause/andropause) to comprehensively understand the mechanisms involved.

## Conclusion

**Figure 7:**
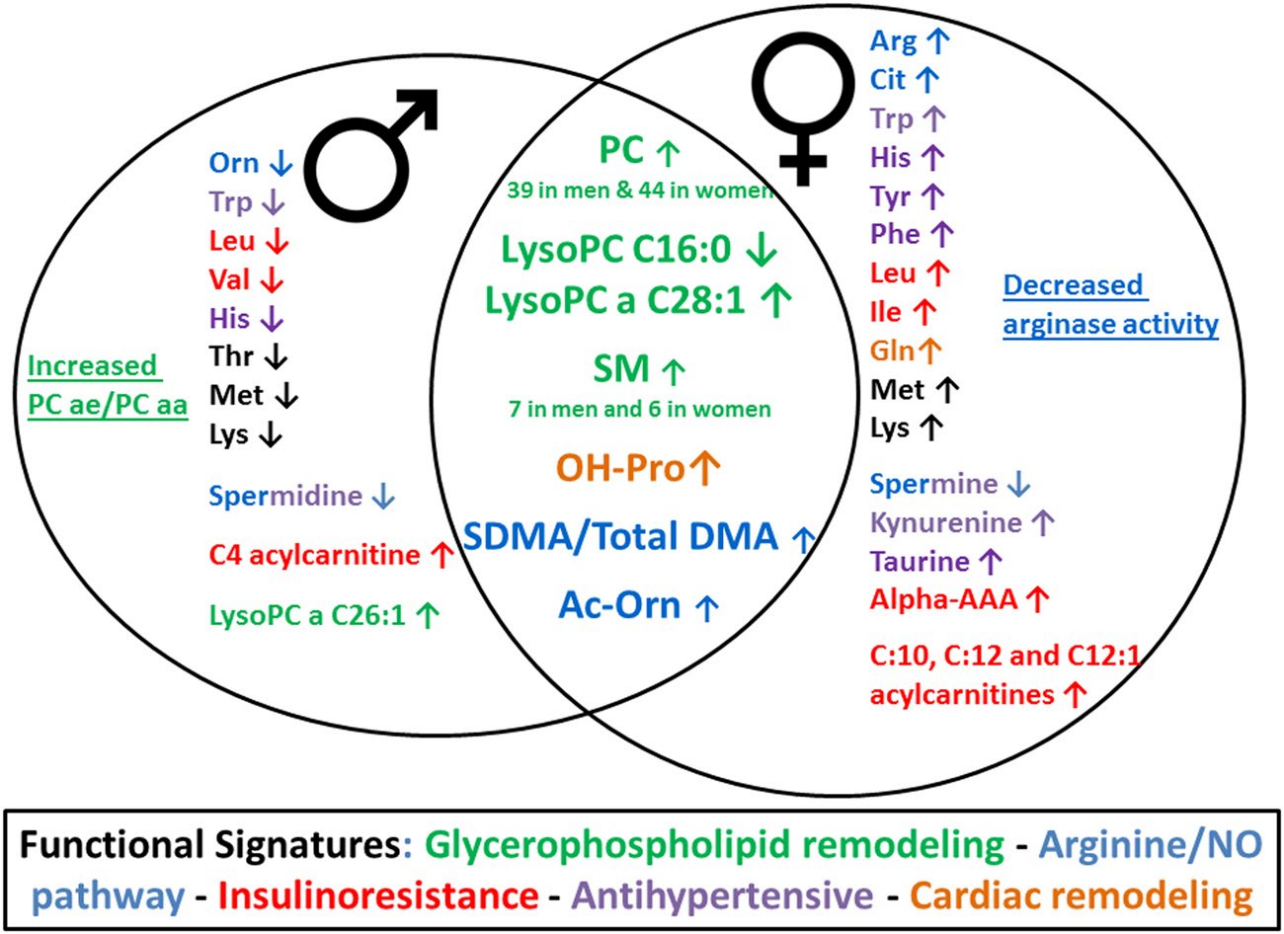
Integrative model of the common and sex-specific signatures and their main functional significance.

In total, Fig. 7 provides an integrative view according to the sex of the global hypertension metabolomic signature and its functional consequences. It shows that increased levels of glycerophospholipids, sphingomyelins, hydroxyproline, SDMA, and acetyl-ornithine are highly superimposable between the two sexes. In contrast, the sexual dimorphism is related to various amino acids, biogenic amines, acylcarnitines and lysophosphatidylcholine variations. Functionally, five main pathophysiological mechanisms are highlighted by the signature. Glycerophospholipid and cardiac remodeling equally affect women and men, while insulin resistance and increased antihypertensive mediators affect women almost exclusively. However, altered arginine/NO pathway shows both similarities and specificities according to sex.

## Supplementary information

Supplementary Tables 1, 2 and 3.

## References

[1] Poulter NR, Prabhakaran D, Caulfield M (2015). Hypertension. Lancet..

[2] Zhou B (2017). Worldwide trends in blood pressure from 1975 to 2015: a pooled analysis of 1479 population-based measurement studies with 19.1 million participants. NCD Risk Factor collaboration. Lancet..

[3] Fowkes FG (2013). Comparison of global estimates of prevalence and risk factors for peripheral artery disease in 2000 and 2010: a systematic review and analysis. Lancet..

[4] Amah G, Lévy BI (2007). Particularités de l’hypertension artérielle du sujet noir-africain. Sang Thrombose Vaisseaux (STV)..

[5] Adeloye D, Basquill C (2014). Estimating the prevalence and awareness rates of hypertension in Africa: a systematic analysis. PLoS One..

[6] Bâ HO (2018). Hypertension and Associated Factors in Rural and Urban Areas Mali: Data from the STEP 2013 Survey. Int J Hypertens..

[7] Bosu WK, Aheto JMK, Zucchelli E, Reilly ST (2019). Determinants of systemic hypertension in older adults in Africa: a systematic review. BMC Cardiovasc. Disord..

[8] Spence JD, Rayner BL (2018). Hypertension in Blacks Individualized Therapy Based on Renin/Aldosterone Phenotyping. Hypertension..

[9] Arnett DK, Claas SA (2018). Omics of Blood Pressure and Hypertension. Circ. Res..

[10] Tzoulaki I, Iliou A, Mikros E, Elliott P (2018). An Overview of Metabolic Phenotyping in Blood Pressure Research. Curr. Hypertens. Rep..

[11] Holmes E (2008). Human metabolic phenotype diversity and its association with diet and blood pressure. Nature..

[12] Menni C (2015). Metabolomic identification of a novel pathway of blood pressure regulation involving hexadecanedioate. Hypertension..

[13] Wang L (2015). Reconstruction and analysis of correlation networks based on GC-MS metabolomics data for young hypertensive men. Anal. Chim. Acta..

[14] Dietrich S (2016). Identification of Serum Metabolites Associated With Incident Hypertension in the European Prospective Investigation into Cancer and Nutrition-Potsdam Study. Hypertension..

[15] Ameta K (2017). Essential hypertension: A filtered serum based metabolomics study. Sci. Rep..

[16] Zhao H (2018). Identification of essential hypertension biomarkers in human urine by non-targeted metabolomics based on UPLC-Q-TOF/MS. Clin. Chim. Acta..

[17] Hernández-Aguilera A (2018). Plasma Energy-Balance Metabolites Discriminate Asymptomatic Patients with Peripheral Artery Disease. Mediators Inflamm..

[18] Kulkarni H (2013). Plasma lipidomic profile signature of hypertension in Mexican American families: specific role of diacylglycerols. Hypertension..

[19] Ke C, Zhu X, Zhang Y, Shen Y (2018). Metabolomic characterization of hypertension and dyslipidemia. Metabolomics..

[20] Loo RL, Zou X, Appel LJ, Nicholson JK, Holmes E (2018). Characterization of metabolic responses to healthy diets and association with blood pressure: application to the Optimal Macronutrient Intake Trial for Heart Health (OmniHeart), a randomized controlled study. Am. J. Clin. Nutr..

[21] Chen L (2019). Sodium Reduction, Metabolomic Profiling, and Cardiovascular Disease Risk in Untreated Black Hypertensives. Hypertension..

[22] Hu C (2011). Application of plasma lipidomics in studying the response of patients with essential hypertension to antihypertensive drug therapy. Mol. Biosyst..

[23] Kim M, Jung S, Kim SY, Lee SH, Lee JH (2014). Prehypertension-associated elevation in circulating lysophosphatidlycholines, Lp-PLA2 activity, and oxidative stress. PLoS One..

[24] Hiltunen TP, Rimpelä JM, Mohney RP, Stirdivant SM, Kontula KK (2017). Effects of four different antihypertensive drugs on plasma metabolomic profiles in patients with essential hypertension. PLoS One..

[25] Colafella KMM, Denton KM (2018). Sex-specific differences in hypertension and associated cardiovascular disease. Nat. Rev. Nephrol..

[26] Ahmed S, Hu R, Leete J, Layton AT (2019). Understanding sex differences in long-term blood pressure regulation: insights from experimental studies and computational modeling. Am. J. Physiol. Heart Circ. Physiol..

[27] Benjamin E (2017). Heart Disease and Stroke Statistics - 2017 Update: A Report From the American Heart Association. Circulation..

[28] Tadic M, Cuspidi C, Grassi G, Ivanovic B (2019). Gender-specific therapeutic approach in arterial hypertension - Challenges ahead. Pharmacol. Res..

[29] Leruez S (2018). P. A plasma metabolomic signature of the exfoliation syndrome involves amino acids, acyl-carnitines and polyamines. Invest. Ophthalmol. Vis. Sci..

[30] Leruez S (2018). The metabolomic signature of glaucoma points to mitochondrial dysfunction, senescence and polyamines deficiency. Invest. Ophthalmol. Vis Sci..

[31] Eriksson, L. *et al*. Multi- and megavariate data analysis – Part I: Basic principles and applications. (MKS Umetrics AB, editor. Umea), (2006).

[32] Graessler J (2009). Top-down lipidomics reveals ether lipid deficiency in blood plasma of hypertensive patients. PLoS One..

[33] Brites P, Waterham H, Wanders R (2004). Functions and biosynthesis of plasmalogens in health and disease. Biochim. Biophys. Acta..

[34] Au A, Cheng KK, Wei LK (2017). Metabolomics, Lipidomics and Pharmacometabolomics of Human Hypertension. Adv. Exp. Med. Biol..

[35] Romanowicz L, Bankowski E (2009). Preeclampsia-associated alterations in sphingolipid composition of the umbilical cord artery. Clin. Biochem..

[36] Dobierzewska A, Soman S, Illanes SE, Morris AJ (2017). Plasma cross-gestational sphingolipidomic analyses reveal potential first trimester biomarkers of preeclampsia. PLoS One..

[37] Dorrance AM, Graham D, Webb RC, Fraser R, Dominiczak A (2001). Increased membrane sphingomyelin and arachidonic acid in stroke-prone spontaneously hypertensive rats. Am. J. Hypertens..

[38] Hanamatsu H (2014). Altered levels of serum sphingomyelin and ceramide containing distinct acyl chains in young obese adults. Nutr. Diabetes..

[39] Lana A (2019). Urinary Metabolic Signature of Primary Aldosteronism: Gender and Subtype-Specific Alterations. Proteomics Clin. Appl..

[40] Simko, F. *et al*. Effect of Ivabradine on a Hypertensive Heart and the Renin-Angiotensin-Aldosterone System in *L*-NAME-Induced Hypertension. *Int*. *J. Mol. Sci*. **19** (2018). 10.3390/ijms19103017 PMC6212851 30282928

[41] Achan V (2003). Asymmetric dimethylarginine causes hypertension and cardiac dysfunction in humans and is actively metabolized by dimethylarginine dimethylaminohydrolase. Arterioscler. Thromb. Vasc. Biol..

[42] Veldink H (2013). Effects of chronic SDMA infusion on glomerular filtration rate, blood pressure, myocardial function and renal histology in C57BL6/J mice. Nephrol. Dial. Transplant..

[43] Bode-Böger SM (2006). Symmetrical dimethylarginine: a new combined parameter for renal function and extent of coronary artery disease. J. Am. Soc. Nephrol..

[44] Tain, Y. L. & Hsu, C.N. Toxic Dimethylarginines: Asymetric Dimethylarginine (ADMA) and Symetric Dimethylarginine (SDMA). *Toxins* (Basel). **9** (2017).

[45] Schlesinger S, Sonntag SR, Lieb W, Maas R (2016). Asymmetric and Symmetric Dimethylarginine as Risk Markers for Total Mortality and Cardiovascular Outcomes: A Systematic Review and Meta-Analysis of Prospective Studies. PLoS One..

[46] Potočnjak I (2018). Serum concentrations of asymmetric and symmetric dimethylarginine are associated with mortality in acute heart failure patients. Int. J. Cardiol..

[47] Zheng HK (2018). Metabolic reprogramming of the urea cycle pathway in experimental pulmonary arterial hypertension rats induced by monocrotaline. Respir. Res..

[48] Eisenberg T (2016). Cardioprotection and lifespan extension by the natural polyamine spermidine. Nat. Med..

[49] Toba H (2010). Oral L-histidine exerts antihypertensive effects via central histamine H3 receptors and decreases nitric oxide content in the rostral ventrolateral medulla in spontaneously hypertensive rats. Clin. Exp. Pharmacol. Physiol..

[50] Sved AF, Fernstrom JD, Wurtman RJ (1979). Tyrosine administration reduces blood pressure and enhances brain norepinephrine release in spontaneously hypertensive rats. Proc. Natl. Acad. Sci. USA.

[51] Mitchell BM, Dorrance AM, Webb RC (2004). Phenylalanine improves dilation and blood pressure in GTP cyclohydrolase inhibition-induced hypertensive rats. J. Cardiovasc. Pharmacol..

[52] Waldron M, Patterson SD, Tallent J, Jeffries O (2018). The Effects of Oral Taurine on Resting Blood Pressure in Humans: a Meta-Analysis. Curr. Hypertens. Rep..

[53] Bartosiewicz J (2017). The activation of the kynurenine pathway in a rat model with renovascular hypertension. Exp. Biol. Med. (Maywood)..

[54] Nagy BM (2017). Importance of kynurenine in pulmonary hypertension. Am. J. Physiol. Lung Cell. Mol. Physiol..

[55] Strand, E. *et al*. Serum Acylcarnitines and Risk of Cardiovascular Death and Acute Myocardial Infarction in Patients With Stable Angina Pectoris. *J. Am. Heart Assoc*. **6** (2017). 10.1161/JAHA.116.003620 PMC5523736 28159823

[56] Wang TJ (2013). 2-Aminoadipic acid is a biomarker for diabetes risk. J. Clin. Invest..

[57] Zhenyukh O (2018). Branched-chain amino acids promote endothelial dysfunction through increased reactive oxygen species generation and inflammation. J. Cell. Mol. Med..

[58] Teymoori F, Asghari G, Mirmiran P, Azizi F (2017). Dietary amino acids and incidence of hypertension: A principle component analysis approach. Sci. Rep..

[59] Scherrer U, Randin D, Vollenweider P, Vollenweider L, Nicod P (1994). Nitric oxide release accounts for insulin’s vascular effects in humans. J. Clin. Invest..

[60] Meyer MR, Clegg DJ, Prossnitz ER, Barton M (2011). Obesity, insulin resistance and diabetes: sex differences and role of oestrogen receptors. Acta Physiol. (Oxf)..

[61] Kaufman JS, Durazo-Arvizu RA, Rotimi CN, McGee DL, Cooper RS (1996). Obesity and hypertension prevalence in populations of African origin. The Investigators of the International Collaborative Study of Hypertension in Blacks. Epidemiology..

